# Artificial Intelligence-Based Non-invasive Differentiation of Distinct Histologic Subtypes of Renal Tumors With Multiphasic Multidetector Computed Tomography

**DOI:** 10.7759/cureus.57959

**Published:** 2024-04-10

**Authors:** Mary R Myers, Chakradhar Ravipati, Vinoth Thangam

**Affiliations:** 1 Radiodiagnosis, ACS Medical College and Hospital, Chennai, IND; 2 Radiodiagnosis, Saveetha Medical College and Hospital, Saveetha Institute of Medical and Technical Sciences (SIMATS) Saveetha University, Chennai, IND

**Keywords:** multidetector computed tomography, hounsfield units, deep neural network, renal cell carcinoma, artificial intelligence

## Abstract

Introduction: With rising cases of renal cell carcinoma (RCC), precise identification of tumor subtypes is essential, particularly for detecting small, heterogenous lesions often overlooked in traditional histopathological examinations. This study demonstrates the non-invasive use of deep learning for Histopathological differentiation of renal tumors through quadriphasic multidetector computed tomography (MDCT).

Patients and methods: This prospective longitudinal study includes 50 subjects (32 males, 18 females) with suspected renal tumors. A deep neural network (DNN) is developed to predict RCC subtypes using peak attenuation values measured in Hounsfield Units (HUs) obtained from quadriphasic MDCT scans. The network then generates confidence scores for each of the four primary subtypes of renal tumors, effectively distinguishing between benign oncocytoma and various malignant subtypes.

Results: Our neural network accurately distinguishes Renal tumor subtypes, including clear cell, papillary, chromophobe, and benign oncocytoma, with a confidence score of 68% with the network’s diagnosis aligning with Histopathological examinations. Our network was also able to accurately classify RCC subtypes on a synthetically generated dataset with 20,000 samples.

Conclusion: We developed an artificial intelligence-based RCC subtype classification technique. Our approach is non-invasive and has the potential to transform the methodology in Renal oncology by providing accurate and timely diagnostic information and enhancing clinical decisions.

## Introduction

According to the International Agency for Research on Cancer (IARC), renal cancer constituted more than 2% of all new cancer diagnoses and 1.8% of cancer-related fatalities worldwide, with a male-to-female ratio of 1.5:1. Over the past few decades, there has been a noticeable rise in the incidence of renal cancer, with estimates suggesting over 430,000 new cases diagnosed across 185 countries globally [1]. According to the 1997 Heidelberg classification, the prevalent subtypes of renal cell carcinoma (RCC) can be classified into conventional, i.e., clear cell renal carcinoma, papillary renal carcinoma, chromophobe renal carcinoma, collecting duct renal carcinoma, and unclassified renal carcinoma. Clear cell RCC is the most common, comprising about 70%-80%, followed by papillary 14%-17% and chromophobe 4%-8%. The remaining subtypes are rare, each with an incidence of ≤1% [2]. Determining the subtype of lesion in a renal tumor is important, given the increasing incidence of such tumors. Unfortunately, identifying tiny heterogenous lesions and their subtypes is challenging as they could be missed in Histopathological examination. Furthermore, each lesion is associated with different therapeutic strategies and prognosis [3]. To this end, recent research has developed non-invasive ways to characterize histologic subtypes of renal masses. Several researchers have directed their attention toward using Imaging characteristics, particularly the level of enhancement observed during multiphasic multidetector computed tomography (MDCT), to differentiate various subtypes of RCC [4-6]. Numerous studies have demonstrated that employing both two and three-phase MDCT protocols has consistently identified a higher degree of enhancement in clear cells compared to other subtypes of RCC [7-11].

The use of artificial intelligence (AI) coupled with medical imaging facilitates precise and rapid diagnosis of disease. In this research, we develop a new deep neural network (DNN)-based technique to predict the RCC subtype from peak attenuation values obtained using multiphasic MDCT. We show that our trained neural network is able to accurately classify the RCC subtype using a synthetically generated dataset as well as a practical test sample acquired in our department.

## Materials and methods

MDCT examination

We performed a prospective longitudinal study for a duration of three months at ACS Medical College and Hospital, Chennai encompassing 50 patients (32 males and 18 females) with suspicion of renal tumor, as they exhibited persistent symptoms including back pain, weight loss, and longstanding hematuria. These patients underwent pre-treatment imaging evaluation by MDCT at a tertiary care center. The patients were examined by MDCT Scanner Somatom Scope 32 slice configuration (Siemens Shanghai Medical Equipment Limited) with the following parameters: Scan type - helical/0.8 sec, Gantry tilt - 0 degrees, Tube settings:120 kVp, 200-250 mAs (depending on patient size), Collimation setting of 16 ×0.6 mm, Pitch value- 0.75 to 1.5 and a reconstructed slice thickness of 2 mm. A variable tube current was sent according to a standardized protocol covering the abdomen from the diaphragm to the Iliac crest. Nonionic contrast agent Omnipaque 350, GE Healthcare, Shanghai, China was administered, adapted to each weight in kg, and was automatically injected at a flow rate of 3 mL/s. The patients were placed in the supine position, and each examination phase was performed during expiration. First, a non-contrast CT scan was performed. Following contrast agent administration, a region of interest (ROI) was placed in the thoraco-abdominal aorta junction, with a trigger set to begin at 150 HU. The corticomedullary phase was scanned at 30-45 after obtaining the attenuation of 150 Hounsfield Unit (HU) in the thoracoabdominal aorta, the nephrographic phase at 70-90 s, and the excretory phase at 300-480 s [5,6]. In each patient, peak enhancement measurements were obtained from the lesion in the unenhanced, corticomedullary, nephrographic, and excretory phases and quantified as HUs [7-15]. These measurements were acquired in each area in the contrast phase to assess the peak attenuation values in our picture archiving and communication system (PACS). The acquired enhancement values were sent into our trained feed-forward DNN that predicts the RCC subtype via softmax activation at the output layer.

Inclusion criteria

Patients referred to the Radiology department for abdominal imaging with suspicion of Renal tumor and those willing to participate in the study with their informed consent signed have been included in the study.

Exclusion criteria

Pregnant females and children less than 18 years old, patients showing allergic reactions to iodine contrast, and patients not willing to participate in the study were excluded.

Ethical approval

This research strictly adhered to the ethical guidelines established in the Declaration of Helsinki and its subsequent amendments. Prior to initiating the study, a comprehensive review was conducted of the study protocol, which included a clear delineation of Objectives, Methodologies, and potential Risks and Benefits for participants. Participants received thorough briefings on the study’s Goals, Methodologies, Risks, and Benefits. Each participant provided written informed consent before engaging in any study-related procedures. It was emphasized that participation was entirely voluntary and participants retained the right to withdraw at any stage without facing adverse consequences. Throughout the study, the confidentiality of personal and medical information was rigorously maintained, with data anonymized to ensure protection and privacy.

Statistical analysis

Demographic features and the peak enhancement values were compared using a one-way analysis of variance (ANOVA). P-value was considered significant if P <0.05. SPSS (version 20.0, IBM Corp., Armonk, NY) was utilized for statistical analyses. We found statistical significance between the enhancement values and their specific correlation to each of the subtypes. To address potential clustering effects, considering that 4 out of 50 patients presented with multiple lesions, the data were also analyzed by including only one lesion per histologic subtype per patient. In cases where multiple lesions were present, the selected lesion was chosen randomly.

Neural network architecture

**Figure 1:**
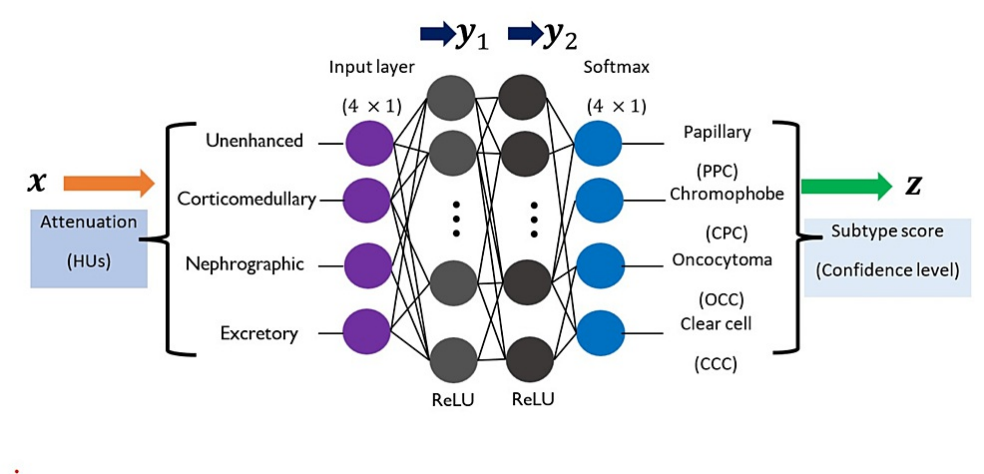
Feed-forward deep neural network-based RCC subtype classifier It is a 4×1 vector *x* containing peak enhancement values at the input layer. The vector *x*, called the Feature vector, is then passed through the first hidden layer, which is defined by a Weight matrix W1 and a Bias vector b1. The output of this layer is defined as y1=ReLU(W1x+b1), where ReLU(⋅) denotes the Rectified Linear Activation Function. We have constructed this neural network independently, drawing upon our background knowledge of machine learning from prior studies done by C.M. Bishop and N.M. Nasrabadi [16].

Our proposed network (Figure 1) is a feed-forward neural network that takes a vector containing peak enhancement values at the input layer. The vector *x* called the Feature vector, is then passed through the first hidden layer, which is defined by a Weight matrix W_1_ and a Bias vector b_1_. The output of this layer is defined as y_1_ = ReLU(W_1_*x* +b _1_), where ReLU(.) denotes the Rectified Linear Activation Function. The function sets negative arguments to zero and it allows positive arguments to pass through, i.e., ReLU(x) =max(x,0).  Also, W_1_*x* is a vector obtained by multiplying the matrix W_1_ with the vector *x* [16].

Next, the vector y_1_ is fed as the input to the next hidden layer, which is defined by a Weight matrix W_2_ and a bias vector b_2_. The output of this layer is defined as y_2_ = ReLU(W_2_y_1_ + b_2_). Finally, the vector y_2_ is passed through the final layer, which is defined by a Weight matrix W_3_ and a Bias vector b_3_. A softmax activation function is applied at the final layer to provide a confidence score vector:

 z = Softmax(W_3_y_2_ + b_3_)

The softmax output vector z is a 4 ^x^ 1 vector comprising likelihoods that x belongs to a particular RCC subtype. The RCC subtypes are considered in the order: of Papillary (PPC), Chromophobe (CPC), Oncocytoma (OCC), and Clear cell (CCC). In this research, we use 50 nodes at each of the two hidden layers. As a result, the matrices W_1_, W_2,_ and W_3_ and have dimensions of 50x4, 50x50, and 4x50, respectively. Furthermore, the bias vectors b_1_, b_2, _and b_3_ are of lengths 50, 50, and 4.

Dataset and training

To the best of our knowledge, there is no publicly available large dataset that has a one-on-one correspondence between the vector of peak enhancements and the RCC subtype. It was also difficult to establish such a dataset in our lab. To address this issue, we synthesized a large dataset using statistical characteristics of the peak enhancements in for each RCC subtype [6,7]. Specifically, the mean and standard deviation of the peak enhancements reported in were used to construct 80,000 samples for each of the four RCC subtypes within our training dataset [6,7].

Using the dataset taken from previous studies by Young et al. and Atul et al. [6,7], 80,000 input feature vectors (x s), each of size 4x1, were constructed according to the normal distribution. This was done using the randn(.) function within the numpy library of python. A similar procedure was carried out for the other entries of the feature vector, and also for all the other subtypes.

In our training dataset, the output labels corresponding to PPC, CPC, OCC, and CCC subtypes were set to the one-hot encoded vectors (1,0,0,0), (0,1,0,0), (0,0,1,0) and (0,0,0,1), respectively. The network was then trained to minimize the cross-entropy loss between the output label and the prediction made by the network, i.e., z. The training was done in Keras using tensorflow as a backend on a python platform. The training procedure optimizes the network parameters, i.e., the weights W_1_, W_2_, W_3_, and the biases b_1_, b_2_, and b_3_.

## Results

Imaging findings

**Figure 2:**
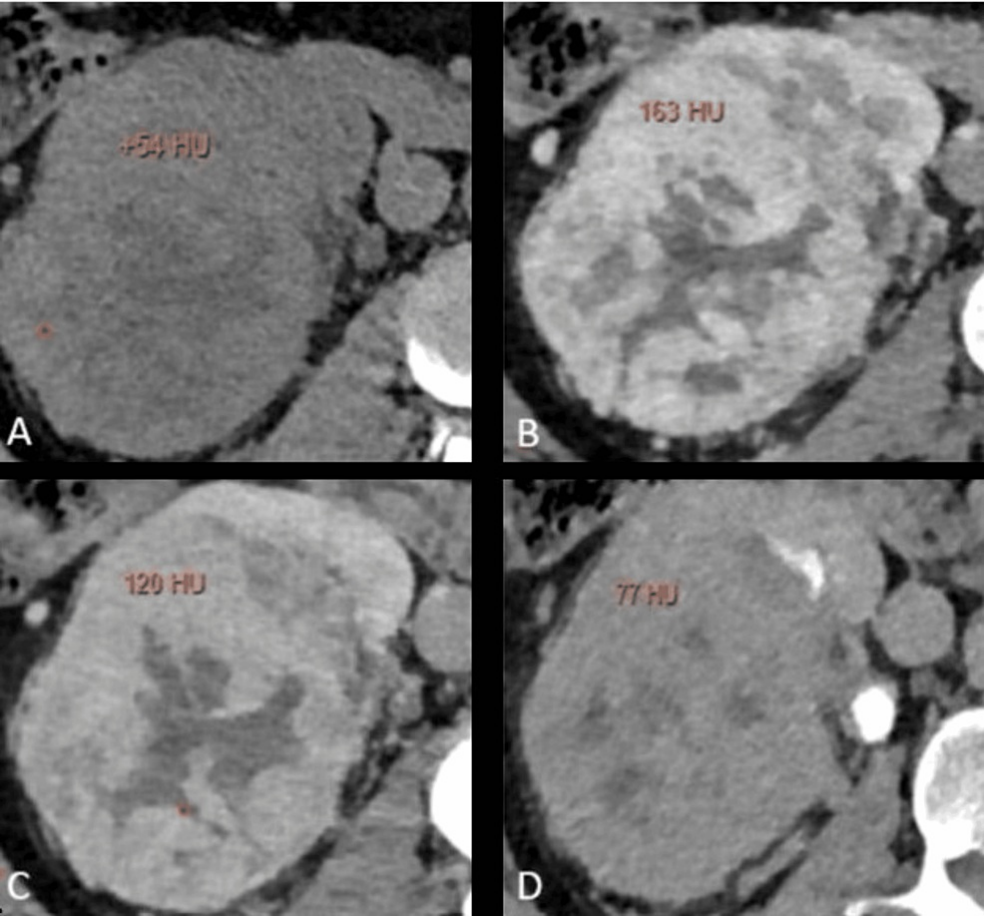
Multiphasic peak attenuation values of clear cell carcinoma (CCC) in a 72-year-old male Axial contrast-enhanced MDCT images depicting the lesion within the right kidney with heterogeneous enhancement, along with corresponding peak enhancement values in (A) unenhanced (+54 HU), (B) corticomedullary (+163 HU), (C) nephrographic (+120 HU), and (D) excretory phases (+77 HU). Attenuation values were measured in Hounsfield Units (HU). These images are our original creation, not copied or sourced from elsewhere. (Photograph courtesy of Dr. Mary Rachel Myers @ 2023 Dr. Mary Rachel Myers, A.C.S Medical College and Hospital, Chennai. All Rights Reserved.)

**Figure 3:**
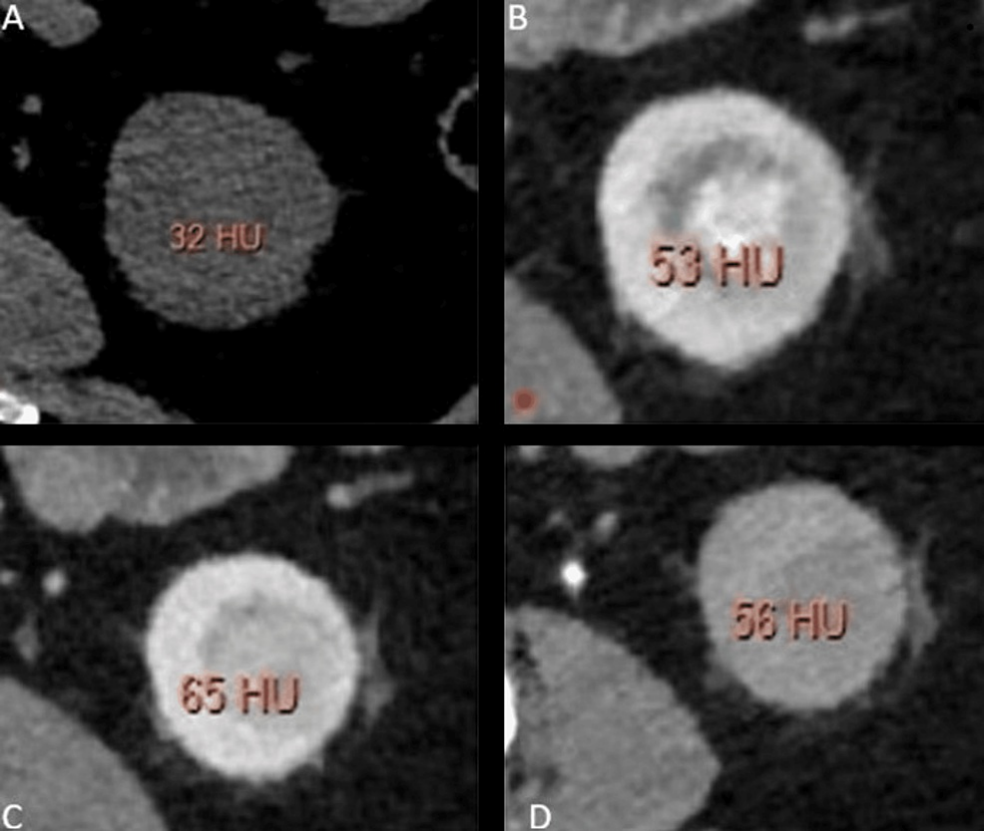
Multiphasic peak attenuation values of Papillary cell carcinoma (PPC)in a 68-year-old female Axial contrast-enhanced MDCT images revealing the lesion within the left kidney with relatively homogenous enhancement, along with corresponding peak enhancement values in (A) unenhanced (+32 HU), (B) corticomedullary (+53 HU), (C) nephrographic (+65 HU), and (D) excretory phases (+56 HU). Attenuation values were quantified in Hounsfield Units (HUs). These images are our original creation, not copied or sourced from elsewhere. (Photograph courtesy of Dr. Mary Rachel Myers @ 2023 Dr. Mary Rachel Myers, A.C.S Medical College and Hospital, Chennai. All Rights Reserved.)

**Figure 4:**
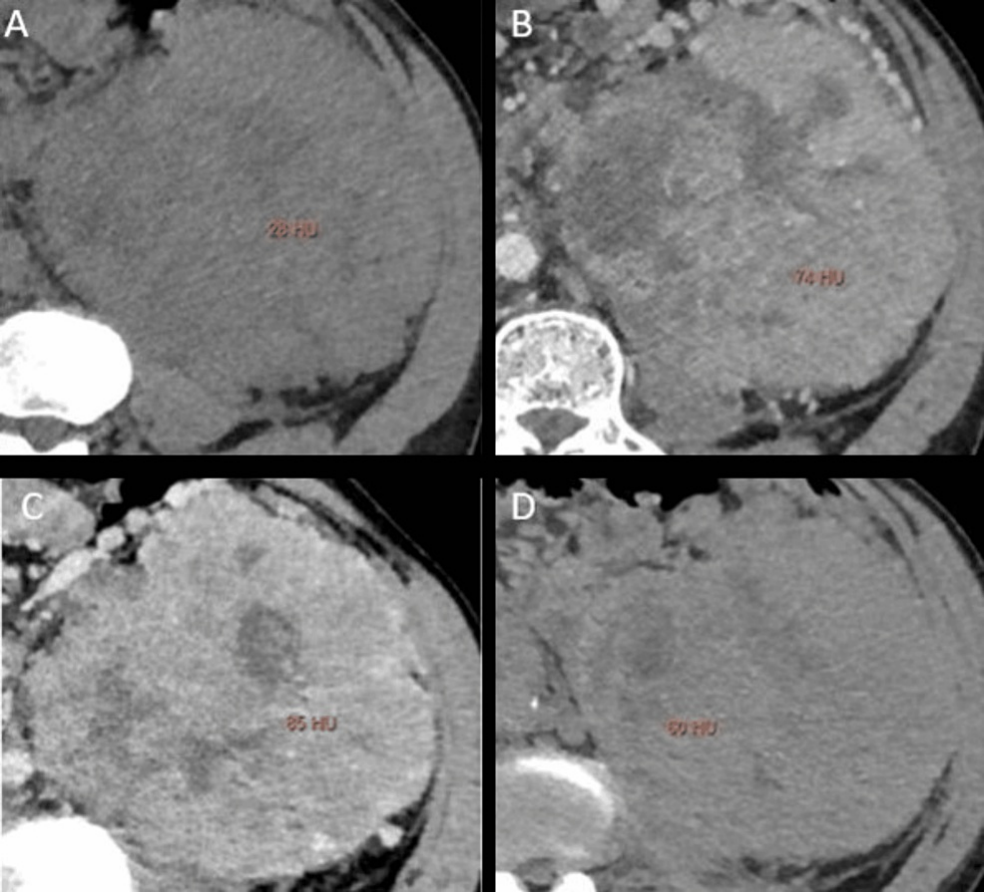
Multiphasic peak enhancement measurements from a 55-year-old male with chromophobe (CPC), confirmed by pathological assessment Axial contrast-enhanced MDCT images depicting the lesion within the Left kidney with heterogenous enhancement, along with corresponding peak enhancement values in (A) unenhanced (+28 HU), (B) corticomedullary (+74 HU), (C) nephrographic (+85 HU), and (D) excretory phase (+60 HU). Attenuation values were measured in Hounsfield Units (HUs). These images are our original creation, not copied or sourced from elsewhere. (Photograph courtesy of Dr. Mary Rachel Myers @ 2023 Dr. Mary Rachel Myers, A.C.S Medical College and Hospital, Chennai. All Rights Reserved.)

**Figure 5:**
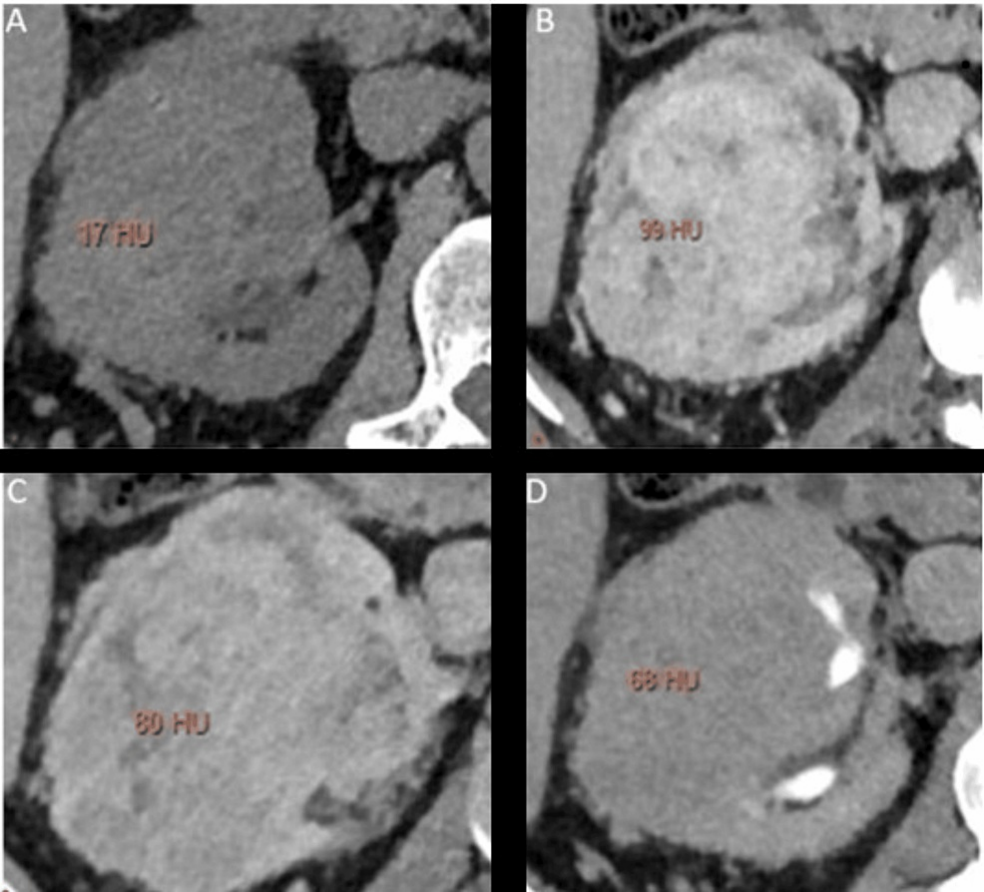
Multiphasic peak attenuation values of oncocytoma (OCC) in a 49-year-old male Axial contrast-enhanced MDCT images depicting the lesion within the right kidney with heterogenous enhancement, along with corresponding peak enhancement values in (A) unenhanced (+17 HU), (B) corticomedullary (+99 HU), (C) nephrographic (+80 HU), and (D) excretory phases (+68 HU). Attenuation values were measured in Hounsfield Units (HUs). These images are our original creation, not copied or sourced from elsewhere. (Photograph courtesy of Dr. Mary Rachel Myers @ 2023 Dr. Mary Rachel Myers, A.C.S Medical College and Hospital, Chennai. All Rights Reserved.)

The enhancement pattern and the peak attenuation of the lesions vary in different phases for various types of lesions, with examples shown in Figures 2-5. The peak enhancement value for clear cell RCCs was significantly greater than that of the other RCC subtypes and oncocytomas.

Proposed DNN-based RCC subtype classification

In this section, we explain our DNN-based RCC subtype classifier that takes a vector with peak enhancement values as the input and outputs confidence scores associated with each of the four RCC subtypes. Specifically, the input vector comprises four peak enhancement values associated with Unenhanced, Corticomedullary, Nephrographic and Excretory phases as the features. A good classifier is one that generates a high confidence score for the true RCC subtype and a low score for the others as shown in Table 1.

**Table 1:** Confusion matrix with our proposed neural network-based classifier Analysis of Renal cell carcinoma (RCC) subtypes indicates varying degrees of predictive accuracy. The matrix illustrates a hierarchy where the highest values correspond to the actual subtype and lower values signify misclassification. The values reported in this table are in percentages. PPC: Papillary RCC, CPC: Chromophobe RCC, OCC: Oncocytoma, CCC: Clear Cell RCC

RCC subtype		Predicted			
(Actual)		PPC	CPC	OCC	CCC
PPC		95.6	4.1	0.1	0.3
CPC		6.5	89.2	0.7	3.7
OCC		0.2	0.9	91.9	7.2
CCC		2.1	10.4	14.5	73

Performance on test dataset

In this research, we tested out our network using two different approaches. With the first approach, we constructed a synthetic dataset with 20,000 test samples generated according to the mean and standard deviations of the peak enhancements obtained from previous study by Young et al. and Atul et al. [6,7]. These 20,000 samples were generated independently of the training set for a fair evaluation. The confusion matrix computed using the predictions derived from the network also indicated high success rates with our proposed classifier. With our second approach, we used the peak enhancement values which were obtained in our lab and projected these inputs into the neural network to arrive at the diagnosis.

Inference

To see how our network can be used in a diagnostic setting, we input the peak enhancement values obtained at each phase, corresponding to each of the patients into the neural network. 

Example 1:  *x*_1_ = (54,163,120,77) was fed into our trained neural network. Such an input results in the output z=(1x10^-3^,8x10^-3^,5x10^-7^,9x10^-2^) at the final layer of our network. As a result, our network diagnoses that the sample corresponds to CCC with a confidence of 99% and this example illustrating the peak enhancement values is shown in Figures 2A-2D. 

Example 2: *x*_2_ = (32,53,65,56) was sent into our trained neural network. The output observed was z=(1.5x10^-1^,8.4x10^-4^,4.0x10^-3^,1.7x10^-4^) which indicates that the sample corresponds to PPC with a confidence score of 89% and the peak enhancement values are depicted in Figures 3A-3D.

Example 3: *x*_3_ = (28,74,85,60) was projected into our trained neural network. The output observed was z =(0.2x10^-3^,1.6x10^-1^,5.7x10^-5^,4.1x10^-3^), which indicates that the sample corresponds to CPC with a confidence of 91% with the peak enhancement values illustrated in Figures 4A-4D.

Example 4: *x*_4_ = (17,99,80,68) was sent into our trained neural network. The output observed was z =(2.3x10^-8^,1.4x10^-5^,8.4x10^-1^,7.6x10^-2^), which indicates that the sample corresponds to OCC with a confidence of 88.9% and the peak enhancement values shown in Figures 5A-5D. 

Our trained neural network mirrored the histopathological examination in 34 out of 50 cases, achieving a confidence score of 68%. This performance signals its potential for reliable use in diagnostic applications. 

## Discussion

In this comprehensive research study, our primary objective was to explore the untapped potential of multiphasic MDCT enhancement as a powerful tool in the realm of renal tumor diagnosis. Specifically, it was used to distinguish between the different subtypes, namely clear cell RCC, papillary RCC, chromophobe RCC, and the benign subtype known as oncocytoma. Our investigation based on deep learning yielded compelling findings that could revolutionize the way we diagnose and manage these distinct renal tumor subtypes.

The study population showed a higher incidence of RCC in male patients (64%) compared to females (32%). In our study, we identified the predominance of CCC accounting for 25 cases (50%), 12 cases (24%) were classified as PCC, five cases as CPC (10%), and eight cases (16%) as OCC as shown in Table 2.

**Table 2:** Demographic characteristics in the present study The table explains incidence of specific type of Renal tumor in the sample population. Among the 50 cases analyzed, Clear Cell Renal Cell Carcinoma (CCC) accounted for the highest incidence at 25 cases, constituting 50% of the sample. Papillary Renal Cell Carcinoma (PPC) followed with 12 cases, comprising 24% of the sample, while (CPC) and Oncocytoma (OCC) accounted for five cases (10%) and eight cases (16%), respectively. CCC: Clear cell carcinoma, PPC: Papillary cell carcinoma, CPC: Chromophobe, OCC: Oncocytoma

Type of Renal Tumor		Incidence (n =50)		
		(%)		
CCC		25 (50%)		
PPC		12 (24%)		
CPC		5 (10%)		
OCC		8 (16%)		

The average lesion size was 3.7 cm for CCC, 3.1 cm for OCC, 2.8 cm for PPC, and 3.5 cm for CPC as illustrated in Table 3.

**Table 3:** Comparison of the average lesion size corresponding to each of subtype of renal tumor The average lesion sizes offer insights into the dimensional differences among renal tumor subtypes. Clear Cell Renal Cell Carcinoma (CCC) had the largest average size at 3.7 cm, while Papillary Renal Cell Carcinoma (PPC) showed a smaller average size of 2.8 cm. Chromophobe (CPC) and Oncocytoma (OCC) had average lesion sizes of 3.5 cm and 3.1 cm respectively, which displays the smallest lesion size.

Type of Renal Tumor		Average lesion size (cms)			
						CCC		3.7			
PPC		2.8			
CPC		3.5			
OCC		3.1			

With the advancement of multiphase CT imaging for renal tumors, including unenhanced, corticomedullary, nephrographic, and excretory phases, we can better distinguish the characteristics of different Renal tumors. This was validated not only in our study but also in studies by Zhang et al., Sheir et al., Kim et al., Ruppert et al., and Chen et al. [10,11,14,15,17], compared to studies like that of Cohan et al. [18], which focused solely on corticomedullary and Nephrographic phases. Vargas et al., research on quantitative multiphase contrast-enhanced MR imaging provides a readily accessible and reproducible method for characterizing various histologic subtypes of Renal tumors. However, it could not facilitate differentiation between clear cell carcinomas and Oncocytomas [19].

In our study, using multiphasic CT imaging, we found a significant elevation of enhancement values for CCC during the corticomedullary, nephrographic, and excretory phases, surpassing those observed in other types of renal tumors. Gakis et al. claimed that oncocytomas exhibited higher enhancement than clear cell RCC in the corticomedullary phase. However, our study and the research conducted by Young et al. and Chen et al. [6,17] disproved this assertion. Notably, while Gakis's study involved only 20 subjects, our study encompassed a relatively larger sample of 50 subjects, offering a more comprehensive basis for drawing conclusions [20].

In quantitative studies, precise evaluation of enhancement patterns is crucial. We advocate for peak enhancement over absolute enhancement measurements, particularly in renal tumor discrimination, due to its simplicity and robustness. Our meticulously designed protocols standardize parameters to optimize peak enhancement evaluation, offering a reliable and clinically relevant method. Compared to previous studies that relied on absolute enhancement measurements to distinguish lesions, meticulous evaluation was imperative. This involves careful consideration of both external and intrinsic factors, as enhancement levels are intricately linked to the amount of contrast material reaching the organ at the moment of measurement. Organ perfusion, including lesions, primarily depends on intrinsic factors such as cardiac function, IV access, patient characteristics, and blood viscosity, but is also influenced by various CT protocol parameters [21-23].

By utilizing multiphasic peak enhancement values our classifier could discriminate between CCC from other subtypes with an accuracy of 76% for this specific subtype (six out of 25 cases of CCC were misdiagnosed as OCC). During our study, the CNN erroneously classified five out of the 12 cases of PCC as CCC, thereby with a diagnostic accuracy of 58.3%. Similarly, two cases of CPC were misinterpreted as OCC, resulting in a diagnostic accuracy of 60%, and miscategorized three cases of OCC as CCC, hereby showing a diagnostic accuracy of 62.5% as shown in Table 4.

**Table 4:** Illustration of accuracy of CNN in classifying specific subtype of renal tumor in the sample size including 50 patients The accuracy of convolutional neural networks (CNN) in classifying Renal tumor subtypes within a sample size of 50 patients is highlighted. Clear Cell Renal Cell Carcinoma (CCC) achieved the highest accuracy at 76.0%, followed by Chromophobe Renal Cell Carcinoma (CPC) and Oncocytoma (OCC) with accuracies of 60.0% and 62.5%, respectively, papillary renal cell carcinoma (PPC) exhibited an accuracy of 58.3%.

Type of Renal Tumor		Accuracy (%)		
		(n=50)		
CCC		76		
PPC		58.3		
CPC		60		
OCC		62.5		

Our results focus on the promising role of multiphasic MDCT, coupled with deep learning-based AI algorithms, in facilitating the discrimination between these diverse renal tumor subtypes. This innovative approach holds great potential to assist radiologists and clinicians in making more informed and timely decisions regarding patient care.

Given the comparatively poorer prognosis and higher likelihood of metastasis associated with CCC in contrast to the other three groups, having a non-invasive method to distinguish between these groups holds significant clinical importance [6]. Unlike invasive procedures, such as biopsy, which carry inherent risks and costs, imaging-based methods offer a safe and cost-effective means of diagnosis. Additionally, this approach allows for the detection of even tiny lesions that may be missed in traditional histopathological examination, ensuring a comprehensive evaluation of the entire tumor.

Furthermore, the clinical implications of our findings in distinguishing lesions such as clear cell RCC and oncocytoma, in particular, exhibit substantially different prognoses and necessitate distinct treatment protocols. It empowers healthcare providers to tailor treatment strategies and surgical approaches, potentially leading to improved patient outcomes and enhanced overall quality of care.

 Discussions limitations of the study

The present study exhibits several limitations. Firstly, a relatively small number of lesion subtypes in the study, warrant further investigation to validate the observed results. Secondly, the results were influenced by the selection of the region of interest (ROI), especially in the case of heterogeneously enhancing lesions, which could introduce Interobserver and Intraobserver bias. Additionally, evaluating the attenuation values of entire renal lesions poses technical challenges that may not be feasible in all clinical settings. It is additionally challenging in instances where there are multiple lesions present in the same patient. The four-phase MDCT renal mass protocol, due to its high patient dose, should be employed judiciously, specifically when lesion discrimination is imperative for treatment decisions.

Recommendations of the study

Despite acknowledging these potential limitations, our analyses point towards a potentially consistent relationship between enhancement observed at MDCT and Histologic characteristics of renal tumors identified by the evolution of AI. Nonetheless, to solidify these findings, validation in a larger cohort, preferably through prospective studies, is essential.

## Conclusions

Drawing from our comprehensive research, we propose that the utilization of quantitative MDCT enhancement patterns exhibits considerable potential in delineating malignant RCC subtypes and benign oncocytoma. Our meticulous analysis underscores the notion that amalgamating AI with these quantitative MDCT enhancement parameters offers a pathway toward the construction of an intricate multiparametric decision model. Such a model, if realized, could emerge as a sophisticated tool augmenting clinical decision-making processes. By providing nuanced insights, it could empower healthcare practitioners to formulate more refined and personalized management strategies, thereby enhancing patient care outcomes in the realm of renal tumors.
